# Versatile Design of Organic Polymeric Nanoparticles for Photodynamic Therapy of Prostate Cancer

**DOI:** 10.1021/acsmaterialsau.3c00060

**Published:** 2023-11-06

**Authors:** Jiacheng Ling, Rongrong Gu, Lulu Liu, Ruixi Chu, Junchao Wu, Rongfang Zhong, Sheng Ye, Jian Liu, Song Fan

**Affiliations:** † Department of Urology, The First Affiliated Hospital of Anhui Medical University, Institute of Urology & Anhui Province Key Laboratory of Genitourinary Diseases, Anhui Medical University, 218 Jixi Road, Hefei 230022, China; ‡ College of Science & School of Plant Protection, Anhui Agricultural University, 130 Changjiang West Road, Hefei 230036, China; § School of Resources and Environment, Anhui Agricultural University, 130 Changjiang West Road, Hefei 230036, China; ∥ Inner Mongolia University Hohhot, Inner Mongolia 010021, China; ⊥ Dalian Institute of Chemical Physics, Chinese Academy of Sciences, 457 Zhongshan Road, Dalian 116023, China; # DICP-Surrey Joint Centre for Future Materials, Department of Chemical and Process Engineering and Advanced Technology Institute, University of Surrey, Guilford, Surrey GU27XH, U.K.

**Keywords:** Photodynamic therapy, Photosensitizer, Organic polymeric nanoparticles, Prostate cancer, Chemotherapy, Photothermal therapy, Reactive oxygen species, Targeting

## Abstract

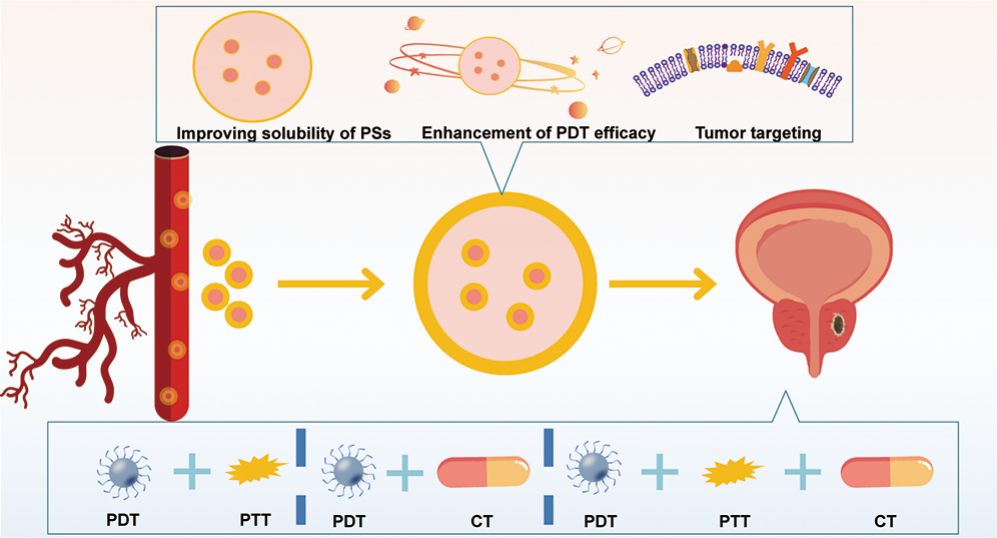

Radical prostatectomy is a primary treatment option for localized prostate cancer (PCa), although high rates of recurrence are commonly observed postsurgery. Photodynamic therapy (PDT) has demonstrated efficacy in treating nonmetastatic localized PCa with a low incidence of adverse events. However, its limited efficacy remains a concern. To address these issues, various organic polymeric nanoparticles (OPNPs) loaded with photosensitizers (PSs) that target prostate cancer have been developed. However, further optimization of the OPNP design is necessary to maximize the effectiveness of PDT and improve its clinical applicability. This Review provides an overview of the design, preparation, methodology, and oncological aspects of OPNP-based PDT for the treatment of PCa.

## 1 Introduction

Prostate cancer (PCa) is the second most common cancer in males.^1^ While localized and regional prostate cancer has a near 100% 5-year survival rate, advanced tumors have a significantly lower survival rate.^2,3^ According to the International Agency for Research on Cancer (IARC) of the World Health Organization (WHO), there will be approximately 1.41 million new cases of prostate cancer and approximately 380,000 deaths worldwide by 2020.^4^ Standard methods for treating prostate cancer often pose significant challenges in clinical practice.^5^ Radical prostatectomy is an option for treating localized disease; however, 20–40% of individuals develop recurrence within 10 years following surgery.^6^ Despite a good initial response, late chemotherapy resistance remains a significant obstacle in clinical practice for the most active chemotherapeutic group of drugs, the paclitaxel class, used to treat metastatic, debulking prostate cancer.^7^ Resistance to traditional therapy and the development of hypoxic zones are characteristics of prostate cancer progression into an advanced stage,^8^ and conventional treatment failure is commonly observed, necessitating the development of alternative treatment strategies.^9^ Hence, to overcome these issues, there has been substantial research on the potential of enhancing treatment through the use of combination medicines and therapeutic techniques that minimize side effects, such as photodynamic therapy (PDT).^10^

PDT has attracted growing attention from researchers due to its noninvasive and effective nature in treating tumors.^11,12^ PDT is a treatment modality that involves the synergistic action of three components: photosensitizer (PS), light, and oxygen.^13,14^ PS, when exposed to light, can generate reactive oxygen species (ROS), which immediately triggers a cascade of events resulting in apoptosis and mitochondrial oxidative damage.^15,16^ By initiating the apoptotic program, the cells may directly induce damage to the target site.

PDT has been employed in the detection and management of prostate cancer and has demonstrated potential advantages in ongoing clinical trials. Partial resection is experienced by 15–50% of patients diagnosed with prostate cancer, and disease recurrence negatively impacts the oncological prognosis.^17^ In contrast, PDT can be performed in a minimally invasive manner by inserting a fiber optic into the target area.^18^ Moreover, the targeting abilities of PSs and the conjunction of PDT with image guidance make it possible to treat tumors effectively and precisely with image-guided photodynamic therapy.^19^ However, the widespread use of PDT in clinical settings has been hindered by factors such as poor clearance of currently approved photosensitizers from the body, low solubility, and low tumor selectivity.^20^ Previous fundamental research has shown that PDT-related treatments using organic polymeric nanoparticles (OPNPs) can overcome these obstacles. On the one hand, OPNPs, which are composed of natural or synthetic organic compounds, are more easily accepted by biological systems. By modifying the surface chemistry and physical properties, they can be made to interact more readily with target cells. Additionally, OPNPs bypass the issue of metal ion-induced toxicity through their NIR absorption capabilities.^21^ On the other hand, encapsulation of PSs in OPNPs improves the solubility and stability of PSs and increases the targeted delivery of PSs into tumors, thereby improving the efficacy of PDT. OPNPs can also be employed to load other therapeutic agents to achieve synergistic therapeutic efficacy. This paper examines the key application strategies, mechanisms of action, and existing experimental data for OPNP-mediated PDT against PCa.

## 2 Principles of PDT

**Figure 1:**
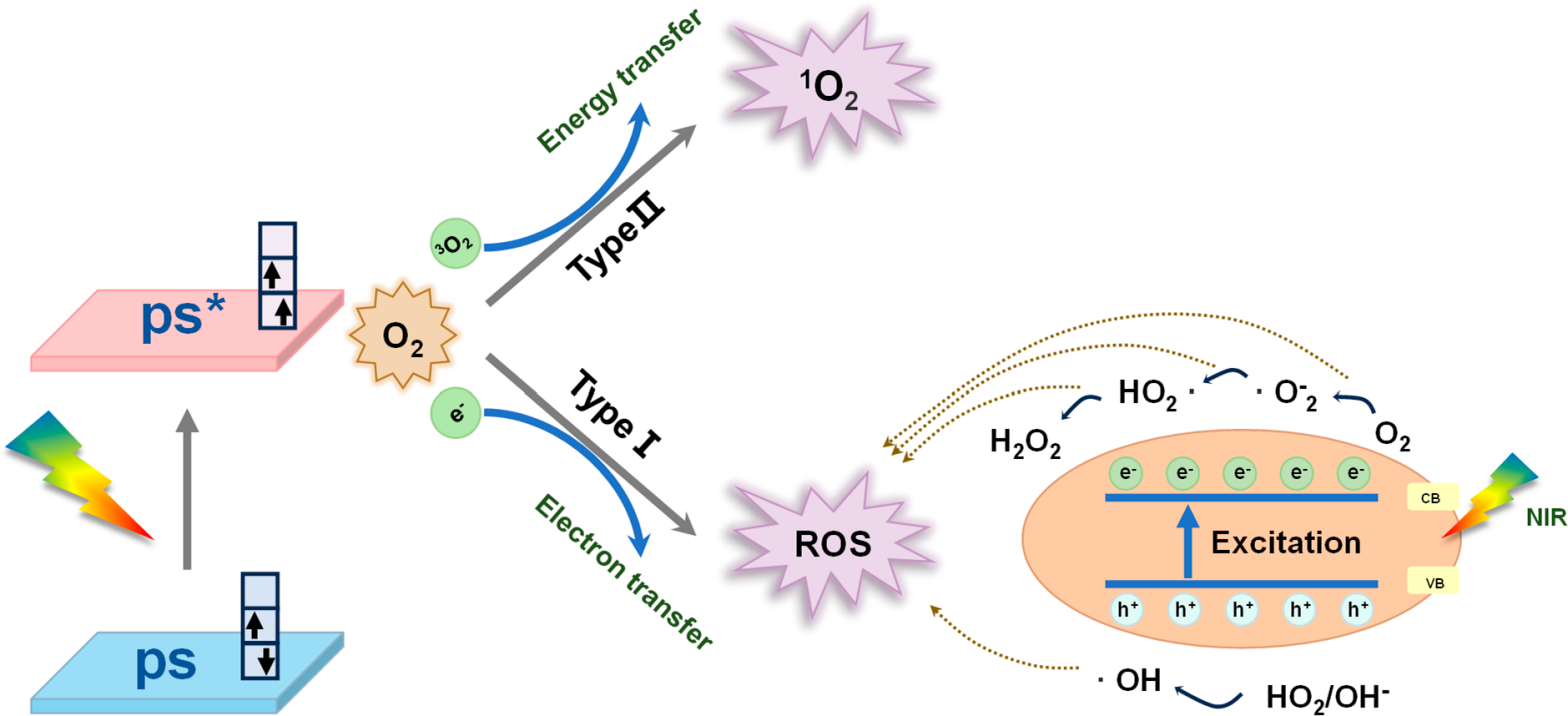
Schematic illustration of photodynamic therapy principle.

The generation of reactive oxygen species is a crucial element of PS-mediated PDT in the presence of light. PDT can be classified into Type I and Type II based on the type of ROS produced by the photosensitizer (Figure 1). Furthermore, PSs induce oxidative stress in PDT, resulting in nonspecific oxidative damage to surrounding biomolecules and thereby exerting cytotoxic effects.^22−24^

### 2.1 ROS-Based Generation of PDT Types

PDT is a nonthermal photochemical reaction involving visible light, oxygen, and PSs as core components.^13^ Intersystem crossing (ISC) is a crucial step in the process of PS-mediated PDT. Upon light exposure (T1), the PS undergoes ISC and transitions to a single heavy state excited state (S1), which has a longer lifetime and generates ROS upon reacting with oxygen or nearby substrates.^25,26^ The types of PDT generation are shown in Figure 1.^27^ In Type I PDT, the photosensitizer transfers its electrons through photochemical reactions to form O_2_^•–^, ^•^OH, or H_2_O_2_, which are weakly reactive and easily react with other molecules that are not susceptible to oxidative damage.^28,29^ In contrast, in Type II PDT, the photosensitizer absorbs light energy and converts its excited triplet state oxygen (O_2_) into excited monomorphic oxygen (^1^O_2_) through energy transfer. ^1^O_2_ is a highly oxidizing ROS that can cause oxidative damage directly or indirectly.^30^ In summary, Type II PDT is the main ROS-producing modality in PDT, with ^1^O_2_ as its main product. On the other hand, Type I PDT generates less reactive ROS and contributes less to the therapeutic effect of PDT. However, Type I PDT is less oxygen-dependent than Type II PDT, and the low-oxygen microenvironment allows for increased PDT potential through intracellular superoxide dismutase (SOD)-mediated disproportionation processes to recycle oxygen and compensate for the amount of oxygen required for PDT.^31,32^ These findings are critical for improving PDT techniques and enhancing clinical efficacy.

### 2.2 Damage of Biomolecules

ROS generated by PDT reacts with various biomolecules such as lipids, proteins, and nucleic acids, leading to the disruption of cellular structure and function. The process of lipid peroxidation occurs as a result of the creation of peroxyl radicals when oxygen and lipids produce lipid hydroperoxides.^33,34^ An excess of ROS leads to the formation of lipid peroxidation,^35,36^ and single oxygen directly incorporates unsaturated lipids to generate lipid oxidation.^35^ By disrupting biological membranes and organelles, including mitochondria, lysosomes, Golgi apparatus, and endoplasmic reticulum, ROS produced by PDT spreads the lipid peroxidation chain reaction, eventually inducing cell death.^37,38^ ROS-mediated protein oxidation mainly modifies cysteine, methionine, tyrosine, histidine, and tryptophan res̀idues.^39,40^ Products of ROS-mediated protein oxidation are mostly determined by side chains, sulfhydryl groups, and amino acid residues. ROS-mediated oxidation of proteins can lead to the hydroxylation of side chains, nitration and sulfation of residues, nitrosylation of sulfhydryl groups, and conversion of some amino acid residues to carbonyl derivatives.^41^ Eventually, ROS-mediated oxidation leads to the cleavage, cross-linking, and aggregation of polypeptide chains,^41^ which disrupts protein structure and denaturation, leading to PDT-mediated cell death.^40,42^ Moreover, PDT-mediated ROS can also damage nucleic acids, leading to the apoptosis of target cells.^43,44^

## 3 Clinical Status of PDT for Prostate Cancer

The prostate is a small, peanut-shaped gland that allows for a high local concentration of medication while avoiding the negative effects and toxicity of surrounding tissue.^45^ Furthermore, compared to other vital organs such as the liver and kidneys, the blood supply to the prostate is relatively modest.^46^ For prostate cancer, PDT can be administered through relatively noninvasive routes, such as the transurethral, transrectal, and perineal routes.^47^ In practical use, PDT can selectively destroy tumor cells and, to some extent, prevent tumor recurrence and metastasis through targeted therapy that combines the architecture of the prostate with noninvasive methods.

### 3.1 Values of Early Studies

Vascular-targeted photodynamic (VTP) therapy is a safe and effective method for treating localized prostate cancer that has not spread to other parts of the body. This treatment involves injecting a light-sensitive drug into the body and then using imaging techniques to guide the delivery of light to the prostate gland. A fiber optic needle is also used to help deliver the light to the prostate.

The first clinical study of PDT for PCa was conducted by Nathan et al.^48^ They gave a light-sensitive drug called *meso*-tetrahydroxyphenyl chlorin to 14 patients and used imaging to place the fiber optic needle. Thirteen of the 14 patients tolerated the treatment well. After treatment, five patients had no visible tumors, and nine had a reduced level of prostate-specific antigen (PSA). In cross-sectional images of the prostate, contrast-enhanced computed tomography or magnetic resonance imaging revealed up to 91% necrosis, or cell death.

Trachtenberg et al. reported on the use of Tookad VTP therapy in humans, showing that it was technically feasible.^3^ They also reported on the effectiveness of Tookad VTP in patients with recurrent limited prostate cancer, confirming its potential as a treatment for this type of cancer.^49^ In another clinical study of VTP therapy for prostate cancer, researchers found that it was well-tolerated and resulted in negative prostate lobe biopsies in most patients who underwent hemiablation.^50^ Azzouzi, Lebdai, and Gill also demonstrated the safety and effectiveness of VTP therapy in clinical trials for low-risk, limited prostate cancer.^51−53^ These studies confirm the value of PDT in achieving controlled tumor necrosis and reducing adverse events for limited disease, local tumor control, and combined minimally invasive treatment.

### 3.2 Limitations of These Studies

**Figure 2:**
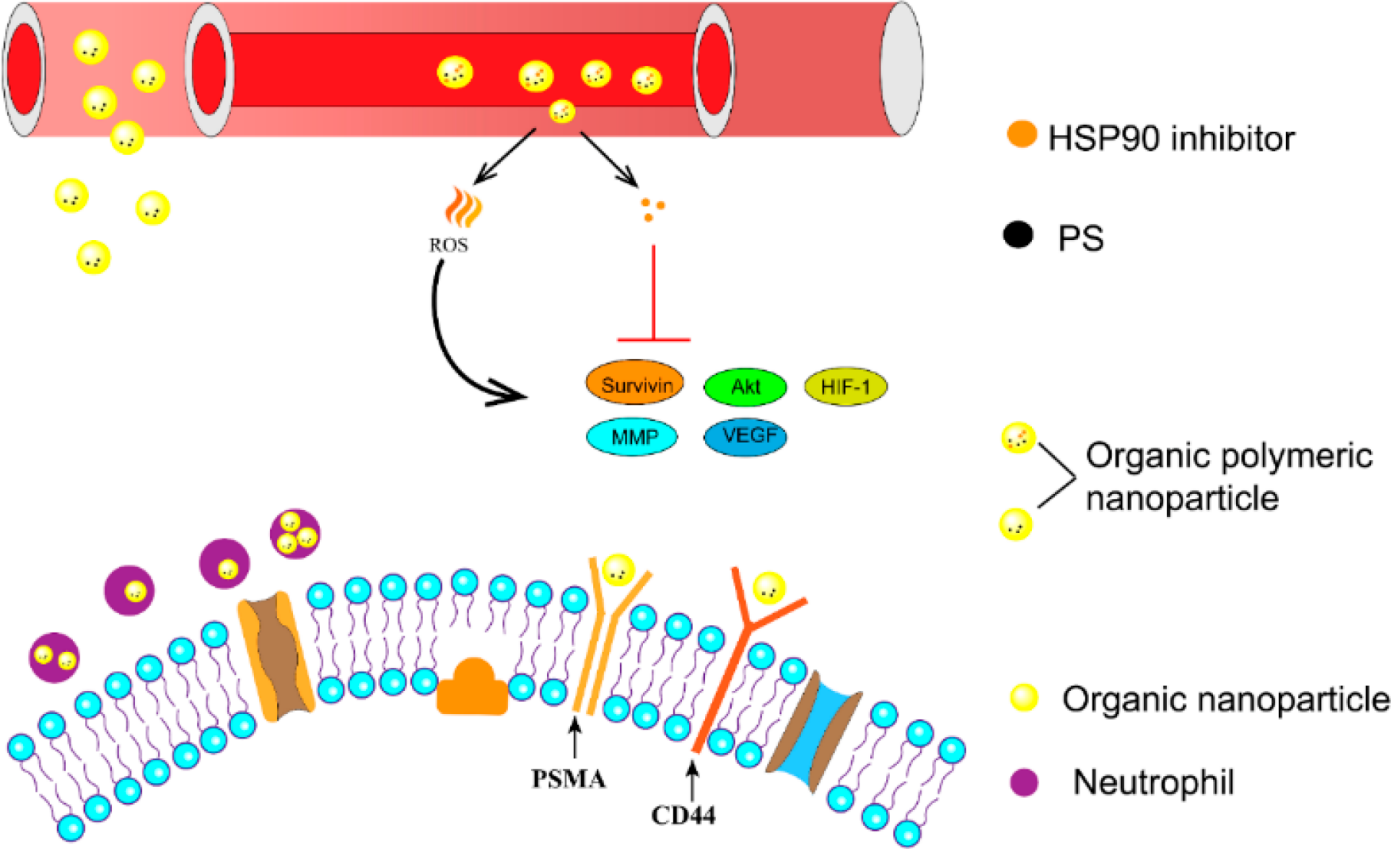
Schematic illustration of PS-loaded OPNPs for localized photodynamic destruction of PCa.

Despite its potential, PDT still has limitations in clinical studies. Some patients have experienced adverse events, such as urethra-rectal fistula and prostatitis, due in part to the poor selectivity of available PSs.^48,49^ The nonselective distribution of PSs can lead to damage to adjacent organs and inefficient accumulation at the tumor site. In some cases, follow-up after PDT has revealed tumor metastases, such as positive biopsies of the liver lobe and the prostate,^50^ which may be due to the low efficiency of the PSs. Common conventional PSs, such as porphyrins and other tetrapyrrole derivatives, are poorly water-soluble and tend to aggregate in physiological solutions because of hydrophobic interactions and π–π stacking.^54,55^ This aggregation-induced burst quenching effect severely impairs PDT efficacy.^56^ In addition, single-mode PDT has limitations in targeting PCa, and in combination with other treatments, such as chemotherapy, is essential to prolong PCa survival.^57−59^ To address these limitations, researchers have developed a range of photosensitizer-loaded OPNPs for both diagnostic and therapeutic investigations in prostate cancer cells. Figure 2 briefly outlines the targeting principles and photodynamic therapy mechanisms of OPNPs in the context of prostate cancer. This paper aims to provide an overview of these concepts and their applications in prostate cancer research.

## 4 OPNPs-Mediated PDT for Prostate Cancer

Local therapy has emerged as a promising approach in the management of prostate cancer due to its reduced side effect profile and comparable efficacy when compared to radical treatment modalities.^60^ Among the various treatment options available, PDT has gained significant interest owing to its high accuracy, minimal side effects, and noninvasive nature. PDT has also been clinically authorized for the treatment of head and neck, pancreatic, and prostate cancers.^61,62^ However, despite its established efficacy for several types of cancer, the low solubility, efficacy, and selectivity of PSs have limited their widespread clinical application in PDT for prostate cancer.^20,63,64^ To overcome this challenge, researchers have explored the use of PS-loaded OPNPs as carriers to selectively deliver PSs to tumor cells for precise and efficient PDT treatment of prostate cancer.

### 4.1 Improving Solubility of PSs

The majority of PSs, including porphyrins and other tetrapyrrole derivatives, are hydrophobic due to the presence of heteroaromatic rings, which promote PS aggregation via hydrophobic interactions and stacking.^54,55^ However, the distinctive construction of PS packages enables high loading capacity, and a well-designed package can prevent fluorescence bursts.^65−67^ For instance, Babic et al. self-assembled 5-ALA-SQ assemblies in an aqueous solution with a 26% loading of 5-ALA.^68^ Similarly, Liang et al. developed a series of PS-loaded OPNPs based on porphyrin grafting lipids (PGL),^67,69,70^ which achieved up to 38.5% porphyrin loading.^67^ These approaches hold promising potential for achieving efficient and targeted delivery of PSs to tumor cells, thereby leading to precise and effective PDT treatment for prostate cancer.

### 4.2 Enhancement of PDT Efficacy

Heat shock protein 90 (Hsp90) is a conserved molecular chaperone that mediates various cellular activities, such as cell transformation, proliferation, and survival under adverse conditions.^71^ As HSP90 is often overexpressed in prostate tumor tissue, it has emerged as a potential target for prostate cancer therapy.^72−74^ Consequently, inhibiting the chaperones that control the HSP90 is a prospective therapeutic approach.^75^ It has strong antiproliferative and cytotoxic effects as well as the ability to degrade proteins.^76^ Recently, it has been suggested that loading Hsp90 inhibitors onto OPNPs could significantly enhance the efficacy of PDT for PCa.

In the study by Lin et al, they developed a nanoporphyrin (OPNP-AAG) that was loaded with an Hsp90 inhibitor for the treatment of prostate cancer.^77^ To achieve this, porphyrin base-terminated dimers were synthesized through solution-phase condensation reactions. The HSP90 inhibitors (such as 17AAG or 17DMAG) were then incorporated into the nanoporphyrin utilizing the “drying method”. Analysis under color transmission electron microscopy revealed that OPNP-AAG exhibited a spherical morphology and retained the structure-dependent fluorescence properties, as well as photodynamic and photothermal conversion properties of empty nanoporphyrins. Moreover, OPNP-AAG was effectively internalized by prostate cancer cells. It was observed that OPNP-AAG predominantly localized in the cytoplasm, displaying a diffuse pattern with multiple dispersed microaggregates. OPNP-AAG played a pivotal role in reducing the levels of pro-survival and angiogenic signaling molecules induced by phototherapy. This, in turn, heightened the sensitivity of cancer cells to phototherapy, ultimately enhancing the photodynamic therapy (PDT) effect on tumors. Furthermore, in vivo studies yielded even more compelling results regarding the treatment efficacy of NP-AAG.

**Figure 3:**
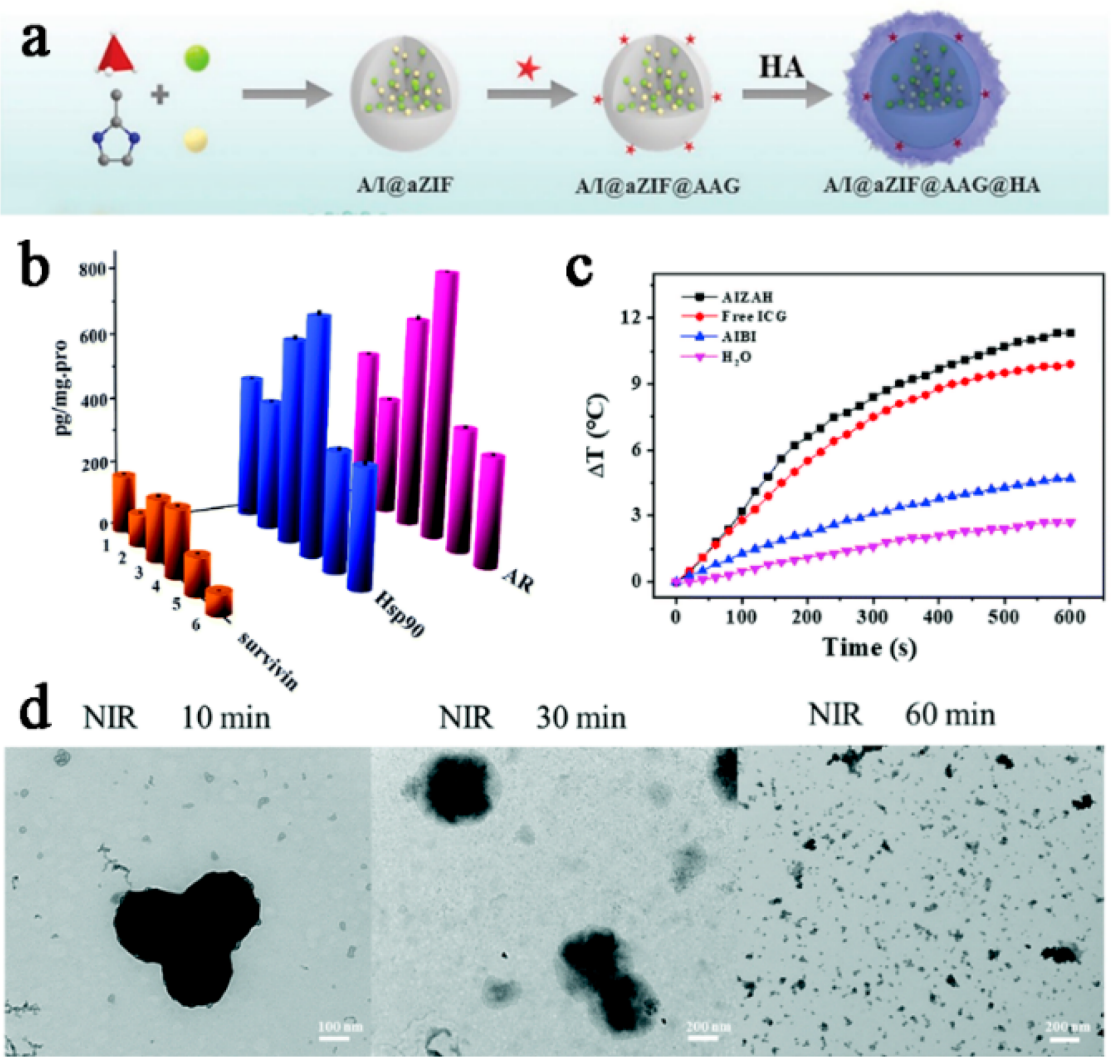
(a) Schematic diagram of the synthesis process of AIZAH OPNPs. (b) Expression of survivin, Hsp90, and AR in LNCaP cells incubated in different treatment groups by ELISA. (c) Temperature rise curve of water, AIBI, free ICG, and AIZAH OPNP solution under 808 nm (1.0 W cm^–2^) near-infrared irradiation for 10 min. (d) TEM images of AIZAH OPNPs with near-infrared (808 nm, 1.0 W cm^–2^) irradiation times of 10, 30, and 60 min at pH 5.0. Reproduced with permission from ref (61). Copyright 2022 Royal Society of Chemistry.

In addition, Sun and colleagues synthesized new multifunctional organic polymeric nanoparticles (AIZAH-OPNPs) for the targeted delivery of the HSP90 inhibitor geldanamycin (17-AAG) via a one-step self-assembly mechanism (Figure 3a).^61^ Compared to the control group, under 808 nm laser irradiation, AIZAH-OPNPs had a favorable photothermal effect (Figure 3c) and achieved inhibition of its receptor proteins, such as the antiapoptotic protein (survivin) and androgen receptor (AR) overexpression, by inhibiting HSP90 (Figure 3b). This enhanced the efficacy of PDT and induced apoptosis in prostate cancer cells. Furthermore, the transmission electron microscopy (TEM) images of AIZAH-OPNPs provided insights into the drug release mechanism. Notably, the degradation rate of the composite material was significantly accelerated following the introduction of thermal stimulation. Specifically, ZIF-8, a key component, exhibited nearly complete degradation within 60 min after exposure to near-infrared (NIR) irradiation (as illustrated in Figure 3d).

### 4.3 Tumor Targeting

OPNPs-based therapeutic approaches are thought to be promising options for tumor diagnosis and therapy, but one of the main obstacles is targeted delivery to tumors.^78^ The accumulation of OPNPs in tumor tissue is facilitated by the enhanced permeability and retention (EPR) effect, which arises from the leaky vasculature and poor lymphatic drainage of tumors.^79,80^ Moreover, OPNPs can be modified to precisely bind to antigens on the surface of cancer cells, which enhances tumor targeting.^81−83^ To improve the efficacy of PDT for PCa, it is crucial to attach PSs to specific ligands or surface receptors of PCa, thereby achieving selective binding and efficient PDT.

#### 4.3.1 Targeting Prostate Tumor Cells Based on PSMA

It is generally known that prostate-specific membrane antigen (PSMA) is a membrane-bound protease that is particular to the prostate. Due to its significant overexpression on malignant prostate tumor cells and its correlation with disease severity, PSMA has been explored as a potential target for detecting and managing PCa.^84^

**Figure 4:**
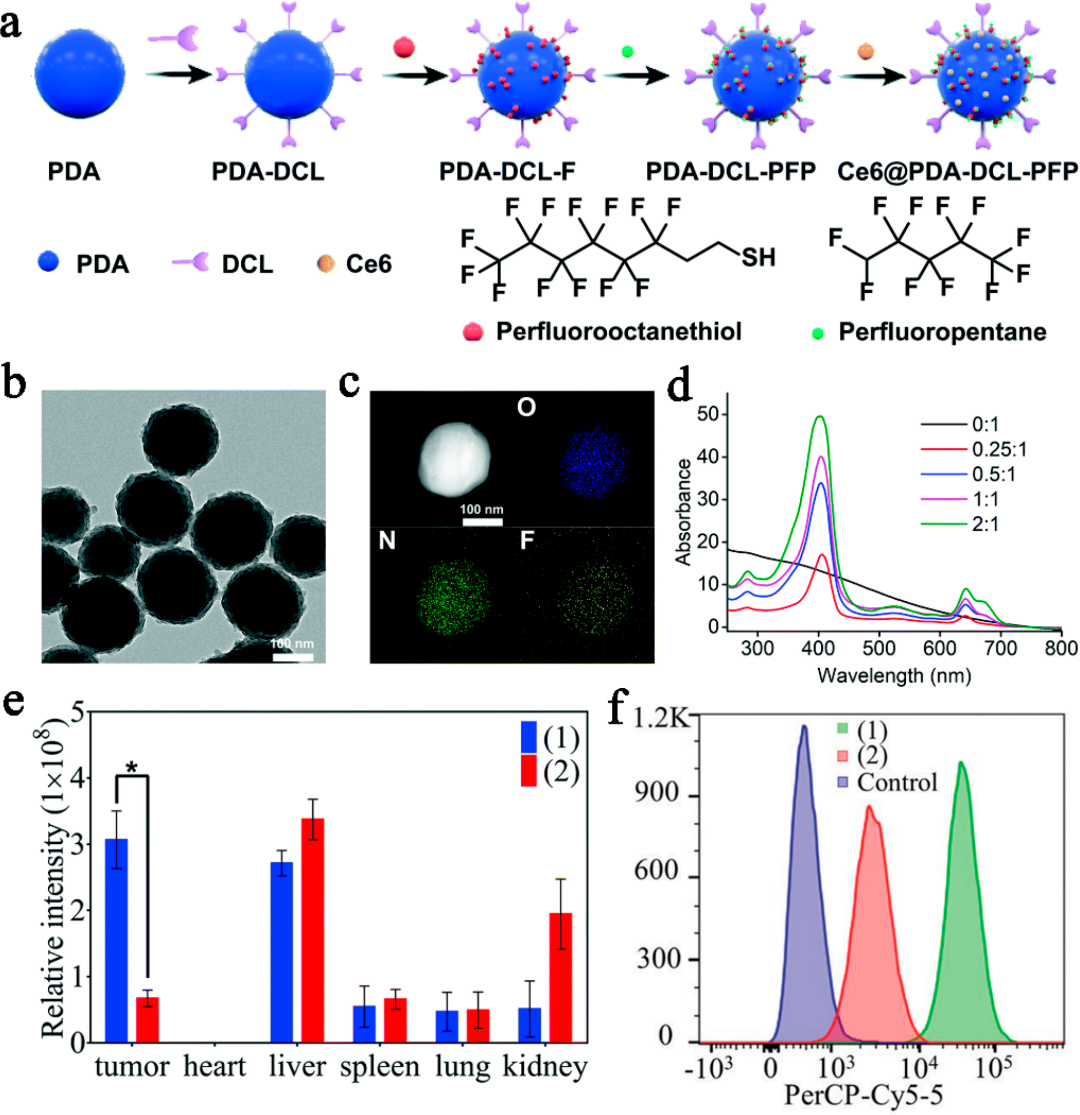
(a) Synthesis of Ce6@PDA-DCL-PFP nanoparticles. (b) TEM of PDA-DCL-PFP nanoparticles. (c) Dark field of vision scanning transmission electron micrographs of single PDA-DCL-PFP nanoparticles show the distribution of O (blue), N (green), and F (yellow). (d) UV–vis spectra of the PDA-DCL-PFP nanoparticles loaded with Ce6 prepared with feed ratios of Ce6 to PDA-DCL-PFP of 0:1, 0.25:1, 0.5:1, 1:1 and 2:1, respectively. (e) Biological distribution of Ce6@PDA-DCL-PFP (group (1)) or Ce6@PDA-PEG-PFP (group (2)) nanoparticles in tumors and organs 24 h after injection was determined by the fluorescence intensity of region-of-interest (ROI). (f) Cell internalization of PSMA positive LNCaP cells on Ce6@PDA-DCL-PFP (group 1) and Ce6@PDA-PEG-PFP (group 2). Reproduced with permission from ref (85). Copyright 2021 Royal Society of Chemistry.

In recent years, PSMA-based targeted OPNPs research has received increasing attention from scholars. Dai et al. synthesized multifunctional melanin-like polydopamine (PDA) OPNPs,^85^ which were modified with the small molecule PSMA inhibitor, *N*-[*N*-[(*S*)-1,3-dicarboxypropyl]carbamoyl]-(*S*)-l-lysine (DCL). The photosensitizer chlorin e6 (Ce6) was loaded onto PDA-DCL after functionalizing it with perfluoropentane (PFP), creating Ce6@PDA-DCl-PFP (Figure 4a). As illustrated in Figure 4d, Ce6 was effectively adsorbed onto the surface of these nanoparticles. TEM images revealed that the PDA-DCL-PFP nanoparticles maintained a spherical shape with an average diameter of 185 nm. Furthermore, there were no discernible morphological changes observed throughout the modification process (as shown in Figure 4b). The scanning TEM map species demonstrated the uniform distribution of N, O, and F in the PDA-DCL-PFP nanoparticles, providing confirmation of their functionalization with PFDT and the loading of PFP on their surfaces (as depicted in Figure 4c). In a comparative analysis with nontargeting probes, it was found that while both were internalized to a similar extent by PSMA-negative LNCaP cells, Ce6@PDA-DCl-PFP exhibited significantly higher cellular uptake (6.5-fold) in vitro and greater tumor aggregation (4.6-fold) in vivo (as shown in Figure 4e, f). These findings indicate a superior capacity for active targeting by Ce6@PDA-DCl-PFP.

#### 4.3.2 Targeting Prostate Tumor Cells Based on CD44

CD44, a highly expressed cell surface glycoprotein in prostate cancer, plays a crucial role in various cellular processes, such as cell–cell interactions, cell proliferation, and cell migration.^86^ Hyaluronic acid (HA) is one of the well-known ligands of CD44. By designing and optimizing OPNPs as a targeted delivery system for CD44 in prostate cancer, the targeting effect of the tumor can be significantly improved, enabling precise treatment of PCa with PDT.

**Figure 5:**
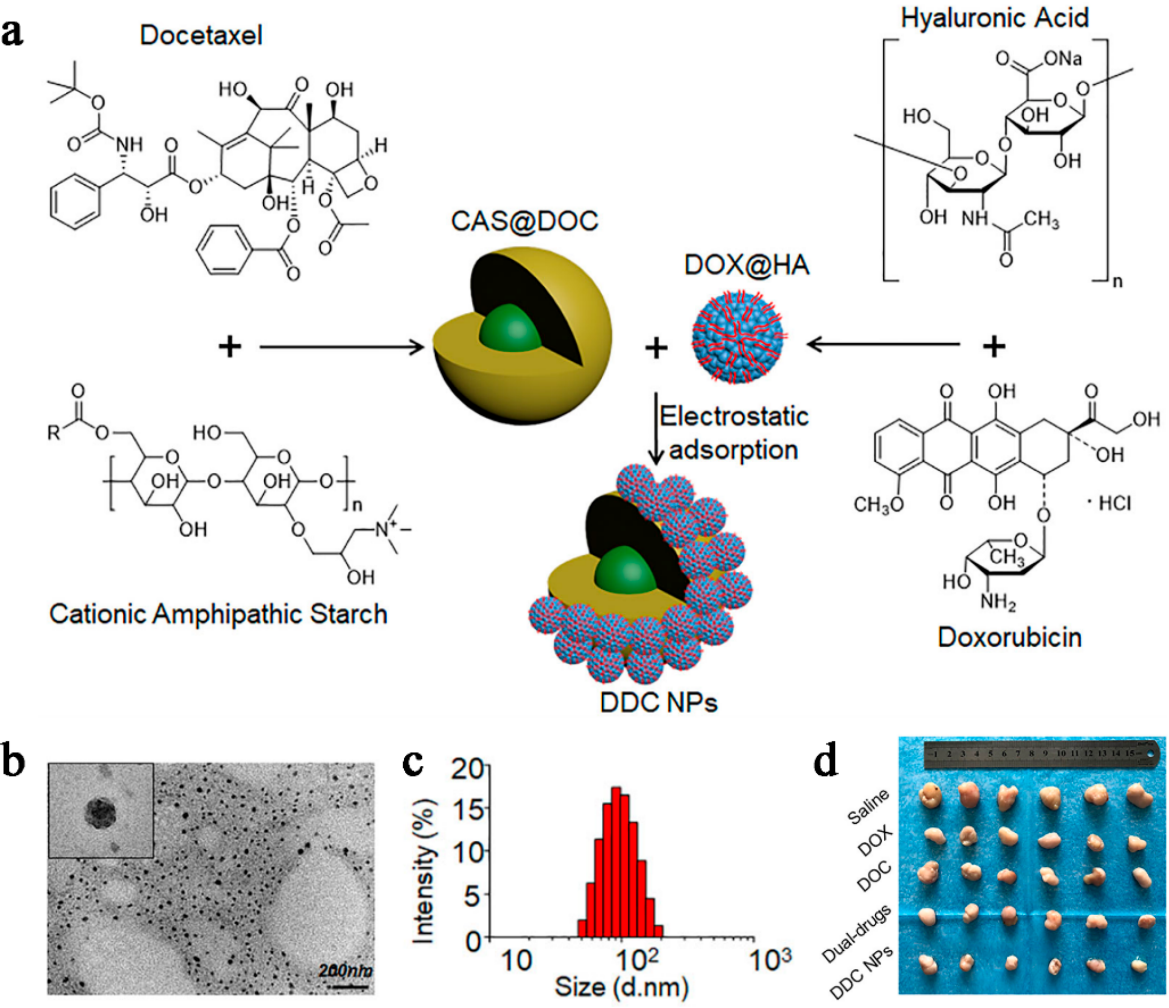
(a) Schematic diagram of the preparation route of DDC OPNPs. (b) TEM images of DDC OPNPs. (c) Size distributions of DDC OPNPs. (d) Optical images of the tumor dissected. Reproduced with permission from ref (87). Reprinted with permission under a Creative Commons [CCBY 4.0]. Copyright 2019 FRONTIERS.

To achieve this, Li and colleagues developed doxorubicin and doxorubicin codelivered organic polymeric nanoparticles (DDC OPNPs) through a self-assembly process (Figure 5a).^87^ TEM images depicted the morphology of DDC OPNPs as spherical particles with rough surfaces. The size and potential results provided further evidence indicating that DOX nanoparticles were adsorbed onto the surface of DOC MCs through electrostatic interactions (as shown in Figure 5b, c). These results collectively demonstrated that DDC OPNPs effectively delivered and fully released the drugs into prostate cancer cells. Additionally, they enhanced drug accumulation within tumors and mitigated the nonspecific aggregation of normal cells by mediating the ligand–receptor interactions between hyaluronic acid (HA) and CD44 protein. Tumor photographs (Figure 5d) revealed that while free drug treatment partially inhibited tumor progression, the inhibitory effect was not satisfactory. Specifically, the average tumor volume increased approximately 6-fold in the DOX treatment group, 6-fold in the DOC treatment group, and 5-fold in the dual-drug treatment group. In contrast, DDC OPNPs exhibited remarkable antitumor effects, with the mean tumor volume increasing only about 2-fold, resulting in significantly smaller tumors compared to the other treatment groups.

**Figure 6:**
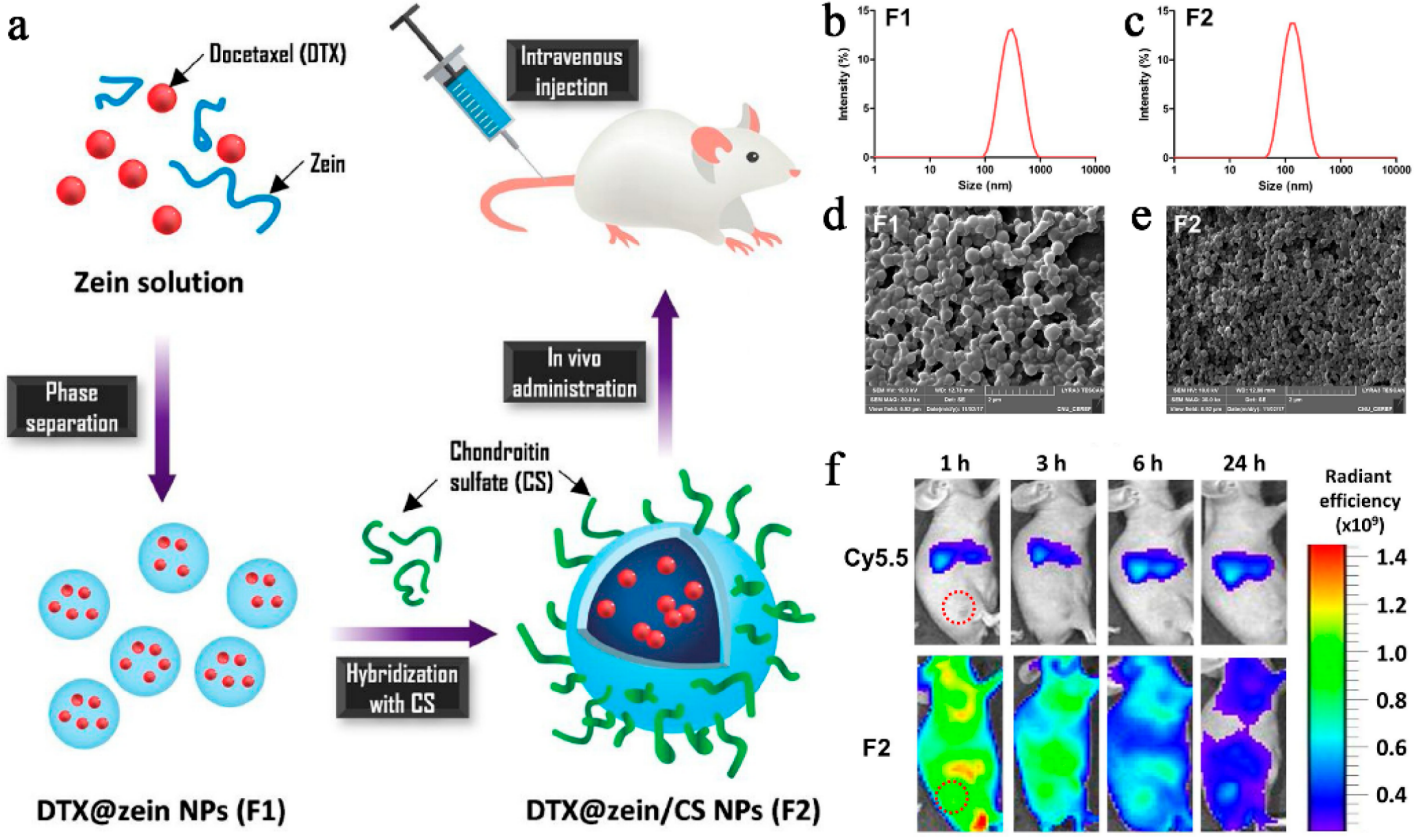
(a) Schematic illustration of DTX@zein/CS OPNPs. (b) Size distributions of DTX@zein OPNPs. (c) Size distributions of DTX@zein/CS OPNPs. (d) SEM images of DTX@zein OPNPs. (e) SEM images of DTX@zein/CS OPNPs. (f) Scan whole body images at 1, 3, 6, and 24 h after injection. Reproduced with permission from ref (88). Copyright 2021 2020 Elsevier Ltd.

**Figure 7:**
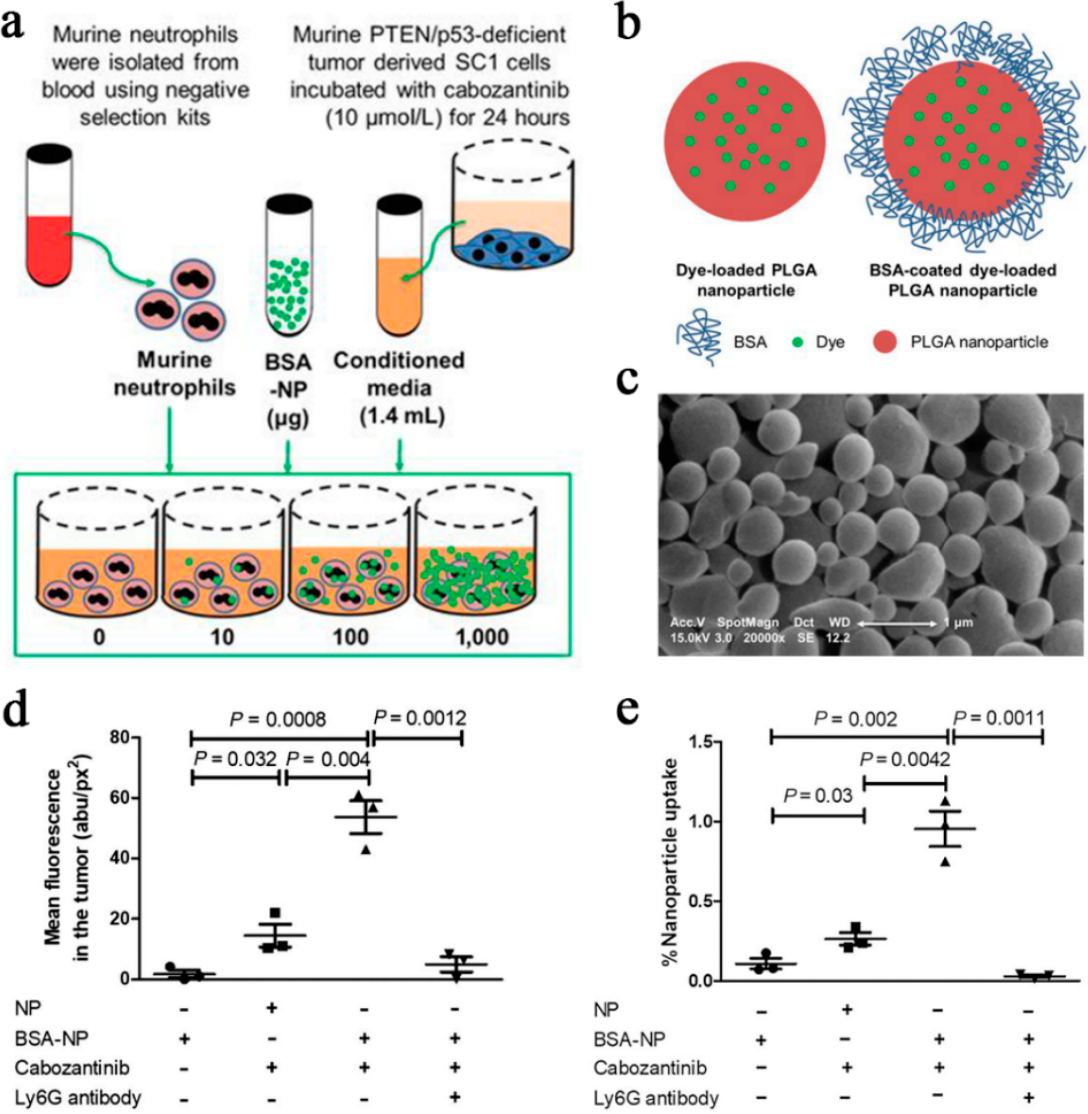
(a) Protocol to evaluate neutrophil function at doses of 10, 100, and 1000 μg BSA-OPNPs. (b) Schematic diagram of dye-loaded PLGA nanoparticles with and without bovine serum albumin coating. (c) SEM images of dye-loaded PLGA nanoparticles. (d) OPNP uptake was assessed by the average fluorescence obtained (*n* = 3). (e) Concentration of DiO-OPNP was determined by the fluorescence method, and percentage of OPNP uptake was calculated according to the dose of OPNPs administered (*n* = 3). Reproduced with permission from ref (93). Copyright 2021 American Association for Cancer Research.

In another study, Lee et al. developed chondroitin sulfate hybridized zeatin organic polymeric nanoparticles (zeatin/CS OPNPs) for the targeted delivery of docetaxel (Figure 6a).^88^ The results presented in Figures 6b and 7c indicate that both Formulation 1 (Fl) and Formulation 2 (F2) exhibit a single-peak size distribution with a polydispersity index (PDI) value of approximately 0.2. Moreover, the inclusion of chitosan (CS) molecules in the formulation enhances the reproducibility of the NP fabrication process. Scanning electron microscopy (SEM) imaging studies further confirm the uniform and spherical morphology observed in both formulations (as shown in Figure 6d and 6e). A solvent displacement technique was employed to produce docetaxel (DTX)-loaded CS-hybridized zein OPNPs at varying zein-to-CS weight ratios. The efficient tumor targeting capability of zein/CS OPNPs was demonstrated through near-infrared fluorescence (NIRF) imaging studies, as depicted in Figure 6f. The enhanced uptake of zein/CS OPNPs by prostate cancer cells can be partially attributed to CD44 receptor-mediated endocytosis, ultimately improving cellular uptake efficiency. These findings collectively suggest that OPNPs designed for CD44 targeting hold significant potential for enhancing the effectiveness of prostate cancer treatment.

#### 4.3.3 Targeting Prostate Tumor Cells Based on Neutrophils

Research has shown that cabozantinib, a small molecule drug that targets tumors, can promote innate immunity against cancer in neutrophils within 72 h.^89,90^ Previous studies have also demonstrated that bovine serum albumin (BSA) can facilitate the internalization of nanoparticles into neutrophils, which can be utilized for targeted delivery to tumor areas.^91,92^ Building on these findings, Chaudagar and colleagues conducted a study to enhance the targeted delivery of BSA-coated organic polymeric nanoparticles (BSA-OPNPs) to the prostate tumor region by inducing neutrophil activation and aggregation using cabozantinib.^93^ To ensure that the internalization of OPNPs by neutrophils does not adversely affect their activation, researchers conducted an experiment in which neutrophils were incubated with varying amounts of DiR-loaded BSA-OPNP (10, 100, and 1000 μg) in 1.4 mL of conditioned medium. The results demonstrated that the binding of BSA-OPNPs did not alter neutrophil activation or function across a range of doses. Moreover, it was observed that OPNP uptake became fully saturated in vivo at a dose equivalent to 100 μg (as shown in Figure 7a). Figure 7b provides dynamic light scattering (DLS) characterization of BSA-OPNPs loaded with DiO and DiR nanoparticles. The DLS analysis revealed an average particle size of 980 nm. Notably, these particles are considerably larger than the BSA-coated PLGA nanoparticles in a previous study, potentially allowing for enhanced drug loading. Furthermore, scanning electron microscopy (SEM) images displayed smooth, spherical particles with minimal aggregation (as depicted in Figure 7c). The results also demonstrated a significant increase of approximately 32-fold in mean fluorescence uptake when cabozantinib/BSA-OPNPs were compared to the control group (Figure 7d). Additionally, the depletion of neutrophils through the use of a Ly6G antibody restored the accumulation of BSA-OPNPs in tumors to baseline levels (Figure 7e).

## 5 Combining PDT with Other Therapies for Prostate Cancer

While PDT shows promise as a treatment option for PCa, further progress is still necessary to improve its efficacy.^94^ PDT alone can actually promote vascular endothelial growth factor, which can facilitate tumor growth and metastasis, thus decreasing its effectiveness. Additionally, PDT has been found to be less effective in treating localized tumors.^95^ To overcome these limitations, various synergistic therapies have been proposed and investigated, including PDT/photothermal therapy (PTT), PDT/CT, and PTT/PDT/CT. These combinations have been shown to induce higher anti cancer efficacy than PDT alone in numerous studies.^96−98^

### 5.1 Combining PDT with PTT

In recent years, PTT, particularly nanomaterial-based PTT, has emerged as a promising approach for the ablation of cancer tumors.^99,100^ PTT works by utilizing a photothermal agent to convert absorbed light energy into heat, which raises the surrounding temperature and causes cancer cells to die.^101,102^ Nanoparticle-based PTT for cancer therapy has notable advantages, such as minimal invasiveness and minor adverse effects.^103^

However, the efficacy of PTT or PDT as single-modality treatments is limited, and the combined use of PDT and PTT has garnered significant attention.^104^ On the one hand, the combined impact of PDT/PTT results in much more potent tumor cell killing than either PTT or PDT alone.^104−106^ On the other hand, PDT can reduce the tumor microenvironment’s ability to protect tumor cells from PTT, and the heat generated by PTT can enhance blood flow and oxygen delivery, improving the efficacy of PDT therapy.^107^ Therefore, PDT/PTT combination therapy has the potential to overcome the limitations of single therapy and improve the effectiveness of oncology treatment.^108^

Dai et al. prepared Ce6@PDA-DCL-PFP OPNPs that demonstrate synergistic effects of PTT and PDT for prostate cancer treatment.^85^ Under 660 and 808 nm irradiation, the nanoparticles showed a synergistic effect of PDT and PTT, inducing a more effective ex vivo killing effect than either treatment alone.

Bhattarai et al. introduced a novel approach for the treatment of prostate cancer, involving the development of anthocyanin-porphyrin combination OPNPs, referred to as PGL-DiR, through a rapid injection method with ultrasonication.^109^ These prepared nanoparticles were designed to enable synergistic PTT and PDT. Transmission electron microscopy images of the nanoparticles provided clear evidence of a mechanism in which the DiR molecule subsequently disintegrated to a size smaller than 50 nm. This transformation was accompanied by a noticeable change in the sample’s color, shifting from green to light red after 10 min of irradiation with a 760 nm laser. These findings underscore the unique switching mechanism of the PGL-DiR nanoparticles, which can be noninvasively modulated using the 760 nm laser. This property makes it a distinctive therapeutic agent for combination phototherapy. In in vivo experiments, it was observed that these nanoparticles, when compared to monotherapies such as PTT or PDT alone, led to a reduction in tumor growth under continuous 760 nm laser irradiation. This discovery holds significant promise for advancing cooperative photothermal nanocarriers in the field of cancer treatment.

### 5.2 Combining PDT with CT

Chemotherapy (CT) is commonly used to treat prostate cancer, but its therapeutic benefits are limited, and it can cause adverse effects.^110^ Even with advanced treatments like androgen biosynthesis inhibition (abiraterone), androgen receptor inhibition (enzalutamide), chemotherapy, or radium-223 combined with androgen deprivation therapy.^111^ noninvasive PDT alone has shown unsatisfactory efficacy in numerous studies.^112−114^ OPNP-based combination treatment, on the other hand, has shown better therapeutic outcomes than PDT or chemotherapy alone. Therefore, an appropriate combination chemotherapy strategy based on PDT is crucial for treating deep tumors, where synergistic effects can be used to target different tumor areas and treat recalcitrant PCa.

**Figure 8:**
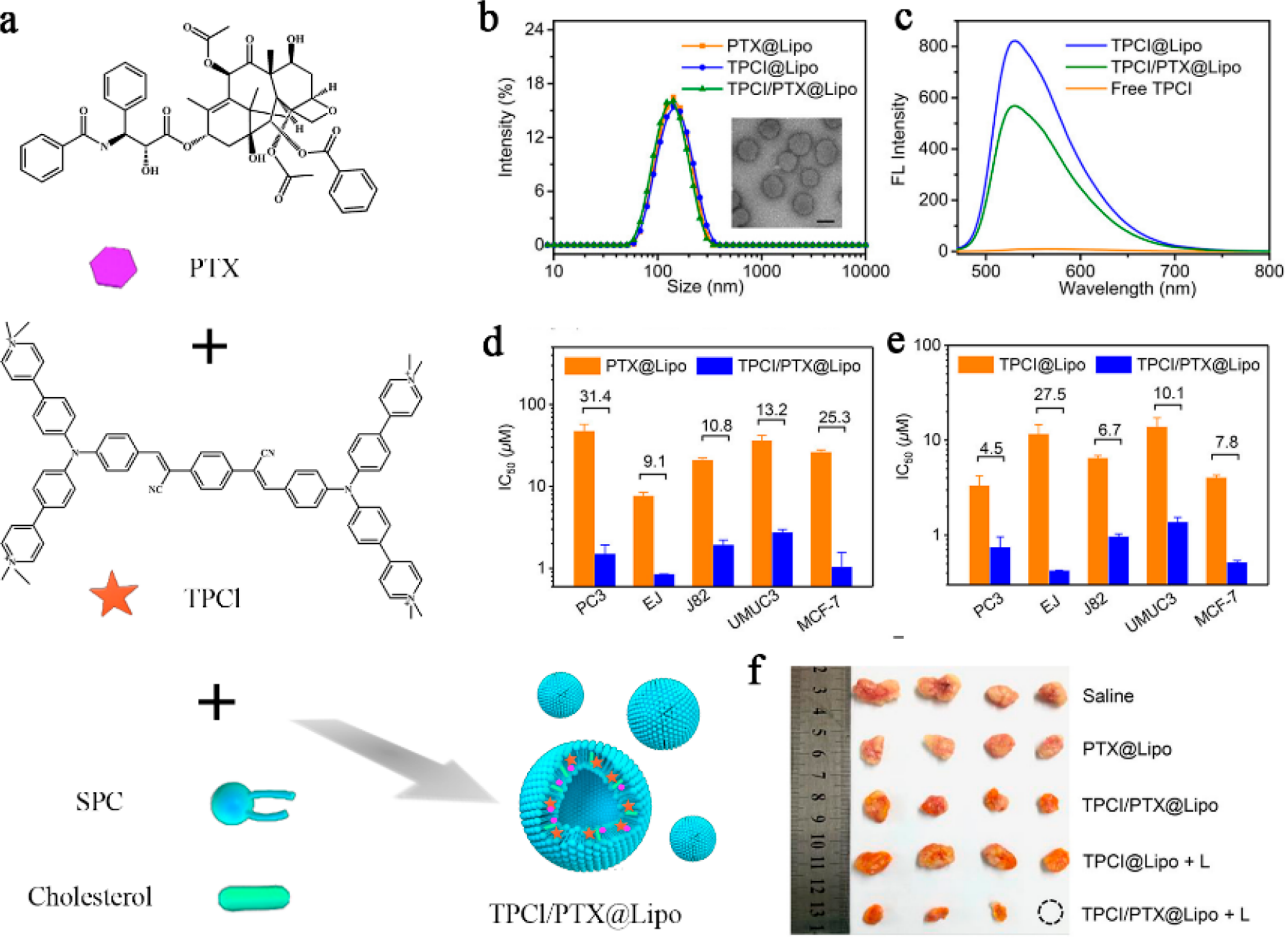
(a) Synthesis of TPCI/PTX@Lipo. (b) Size distributions of PTX@Lipo, TPCI@Lipo, and TPCI/PTX@Lipo. Inset: TEM image of TPCI/PTX@Lipo. (c) Fluorescence spectra of free TPCI, TPCI@Lipo, and TPCI/PTX@Lipo in water. (d) IC50 values of PTX in PTX@Lipo and TPCI/PTX@Lipo in treating PC3, EJ, J82, UMUC3, and MCF-7 (460 nm, 1 mW cm^–2^, 10 min). All data is expressed as mean ± standard variation from three independent experiments (*n* = 5). (e) IC50 values of TPCI in TPCI@Lipo and TPCI/PTX@Lipo in treating PC3, EJ, J82, UMUC3, MCF-7 (460 nm, 1 mW cm^–2^, 10 min). All data is expressed as mean ± standard variation from three independent experiments (*n* = 5). (f) Photograph of tumor tissues on the 14th day after the start of treatment. Reproduced with permission from ref (115). Copyright 2020 American Chemical Society.

A highly efficient photodynamic therapy diagnostic platform (TPCI/PTX@Lipo) was developed by Wang et al.^115^ The platform was synthesized by dissolving phosphatidylcholine and cholesterol (5:1, w/w), a photosensitizer (TPCI), and/or paclitaxel (PTX) in a 1:1 chloroform/methanol mixture, which was then evaporated under vacuum to form a thin lipid film (Figure 8a). The film was hydrated by sonication in a 0.9% sodium chloride solution in an ice bath. As depicted in Figure 8b, the liposomes prepared were spherical in shape, with an average size falling within the range of 100–120 nm. Notably, the fluorescence intensity of TPCI@Lipo and TPCI/PTX@Lipo exhibited a significant increase (as demonstrated in Figure 8c). This enhanced fluorescence can be attributed to the aggregation-induced emission (AIE) effect of TPCI, whereby the intramolecular movement and nonradiative decay of TPCI were inhibited by the presence of phospholipids. Results from in vitro cellular experiments indicated a synergistic effect between PDT and CT, leading to a substantial improvement in treatment efficacy against PC3 prostate tumor cells when compared to PDT or CT alone. This observation underscores a robust synergistic anticancer effect (as depicted in Figure 8d and e). Furthermore, in alignment with these findings, in vivo antitumor studies demonstrated the effectiveness of TPCI/PTX@Lipo OPNPs in eradicating PC3 tumor cells with an initial size of 200 mm^3^ (as shown in Figure 8f).

### 5.3 Multimodal Synergistic Therapy Based on PDT

Multimodal therapies targeting prostate cancer have been continuously explored. Although the powerful absorbance and good biocompatibility of near-infrared (NIR) show exciting potential in nanomedicine, their development in the direction of treating tumors is limited by the existing design approaches.^116^ In recent years, multimodal synergistic therapies based on PDT have received increasing attention in cancer treatment.^117^ The combination of PDT with other therapeutic modalities is emerging as one of the strategies to improve the effectiveness of cancer treatment and minimize side effects.^118,119^ Lian et al. developed multifunctional OPNPs (HSA@IR780@DTX) using IR780, a near-infrared dye, and human serum albumin (HSA)-based docetaxel (DTX) for the multimodal treatment of prostate cancer.^120^ TEM imaging unequivocally confirmed the spherical morphology with a smooth surface for all self-assembled OPNPs. The UV–vis spectra depicted provided clear evidence that the HSA-DTX solution exhibited negligible absorption in the near-infrared (NIR) region. In stark contrast, HSA@IR780 and HSA@IR780@DTX displayed subtly red-shifted absorption peaks when compared to free IR 780. This observed spectral shift can be attributed to modifications in molecular conformation resulting from the intricate binding of IR780 with albumin. To comprehensively evaluate the photothermal properties, researchers meticulously probed the photothermal profiles of IR780 and HSA@IR780@DTX solutions, both of which exhibited analogous photothermal behavior upon exposure to an 808 nm laser. Notably, it was ascertained that the temperature alteration of these nanoparticles was contingent on concentration. In the realm of in vivo efficacy experiments, mice were intravenously administered with either HSA@IR780 or HSA@IR780@DTX. Subsequent laser exposure led to a swift escalation in tumor temperature, reaching approximately 53 °C for mice treated with these nanoparticle formulations. In sharp contrast, mice injected with PBS exhibited negligible alterations under identical laser exposure conditions. Remarkably, prostate tumors in mice subjected to the multifaceted therapeutic regimen involving HSA@IR780@DTX in conjunction with infrared laser irradiation were rendered completely suppressed. Conversely, tumors in mice treated solely with chemotherapy (HSA@DTX and HSA@IR780@DTX without laser) or PTT/PDT in isolation (HSA@IR780 plus laser) exhibited only moderate inhibition of growth.

## 6 Conclusion and Outlook

Photodynamic therapy has demonstrated controlled tumor necrosis and low adverse effects in several clinical studies for nonmetastatic localized prostate cancer, as well as the ability to halt disease progression.^48−53^ However, despite the positive outcomes of earlier clinical trials, PDT is still relatively limited in these clinical research studies. Several preclinical investigations have reported adverse events such as urethra-rectal fistula, prostatitis,^48,49^ and even tumor metastasis in certain individuals.^49^ These issues may be related to the low solubility, selectivity, efficacy, ^1^O_2_ yield, and limited unimodal PDT efficacy of conventional PSs. Therefore, various OPNPs loaded with PSs targeting prostate cancer have been developed to address these issues.

Rationally designed nanocarriers have significantly improved the solubility of PSs in blood under light.^67−70^ By carrying Hsp90 inhibitors, the OPNPs can inhibit the overexpression of their receptor proteins, such as the antiapoptotic protein (survivin) and the androgen receptor (AR), thus improving the efficacy of PDT.^61,77^ By coupling relevant prostate-targeting antibodies, OPNPs can significantly improve the targeting ability of PSs as well as the tumor aggregation effect of PSs.^85^ Various synergistic PDT-based therapies for antitumor efficacy have significantly improved the limited efficacy of PDT alone in treating prostate cancer.^85,109,115,120^ Additionally, imaging-guided PDT with light, involving intravenous injection of photosensitizer and percutaneous insertion of a fiber optic perineural needle into the prostate, has produced good tumor ablation.

**Figure 9:**
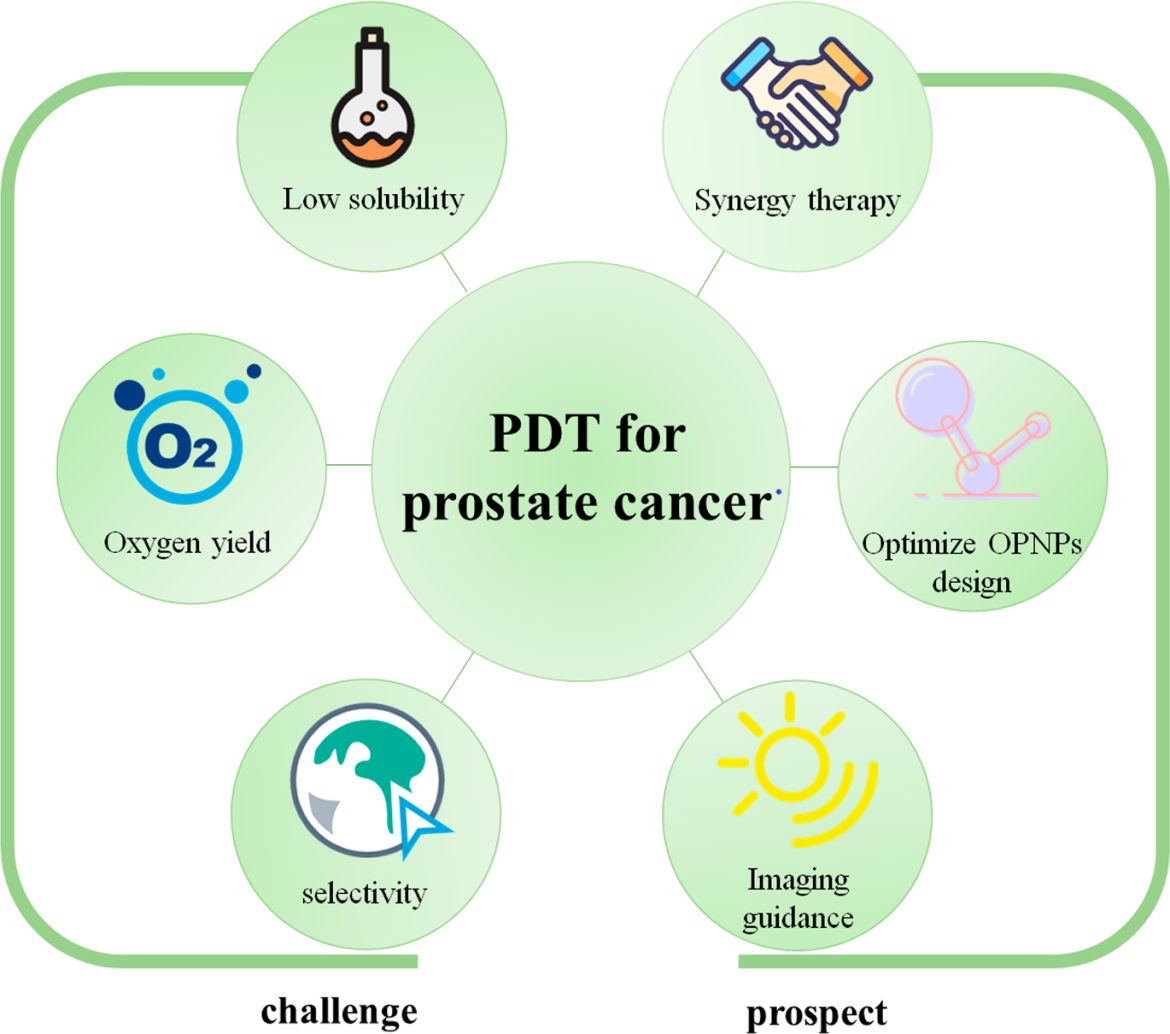
Current challenges and future development of PDT for prostate cancer.

It is crucial to recognize the problems with prostate cancer right now and in the future (Figure 9). While OPNPs loaded with PSs can enhance the production of singlet oxygen by avoiding potential aggregation, reactive oxygen species generation is the essential element of PSs-mediated PDT. Therefore, for maximum PDT efficacy, OPNPs need to be rationally designed to carry more oxygen or to generate oxygen in situ in the prostate tumor region. Additionally, the manufacturing process of OPNPs is more complicated, and the presence of multiple constituents in OPNPs may lead to instability in the control of targeted release and therapy, as well as mutual side effects between multiple components. Although studies have reported the simple and efficient ways in which nanocarriers act as PSs, there are fewer relevant studies in the direction of prostate tumors. Thus, a substantial amount of basic research is required to develop photodynamic nanomaterials for better clinical conversion of OPNP-based PDT. In conclusion, existing OPNP-based PDT has shown promise for the treatment of prostate cancer. However, further research is needed to improve oxygen generation in the tumor microenvironment, optimize OPNP design, and create photodynamic nanomaterials with enhanced efficacy and safety for clinical use.
